# The Origins and Development of Pre-emptive Dermatologic Anesthesia: A Systematic Review

**DOI:** 10.7759/cureus.55851

**Published:** 2024-03-09

**Authors:** Vladislav P Zhitny, Eric Kawana, Benjamin Vachirakorntong, Kenny H Do, Jenifer Do, Ivan Rahman, Nikita S Mehta, Jonathan G Alabre, Aleksandar Kiprovski, Michael C Wajda

**Affiliations:** 1 Department of Anesthesiology, Perioperative Care, and Pain Medicine, New York University Langone Health, New York City, USA; 2 Department of Internal Medicine, Kirk Kerkorian School of Medicine at University of Nevada, Las Vegas (UNLV), Las Vegas, USA; 3 Department of Osteopathic Medicine, Touro University Nevada, Henderson, USA; 4 Department of Life Sciences, University of Nevada, Las Vegas (UNLV), Las Vegas, USA

**Keywords:** general dermatology, anesthesia, perioperative pain management, procedural sedation and analgesia, local analgesia

## Abstract

This study delves into the historical trajectory of dermatological anesthesia, tracing its roots from ancient civilizations to modern times. It emphasizes the relentless pursuit of pain relief in dermatologic procedures and the transformative impact of anesthesia on surgical practices. A comprehensive analysis was conducted through an extensive literature review, employing the Preferred Reporting Items for Systematic Reviews and Meta-Analyses (PRISMA) systematic review model on the PubMed and Embase databases. A total of 1304 articles were initially identified, with six publications from these databases and 10 additional sources from the World Wide Web included in the study. This systematic approach allowed for a thorough examination of the historical journey of dermatological anesthesia. The historical trajectory outlined in this study highlights the progress in dermatological anesthesia, showcasing its impact on contemporary procedures with a continual emphasis on patient comfort and safety. As medical knowledge expands, the ongoing quest for enhanced pain control in dermatology remains a central focus.

## Introduction and background

The history of dermatological anesthesia is deeply intertwined with the history of human suffering and pain, reaching back to ancient civilizations. From ancient Egyptian circumcision procedures to the latest advancements in modern medicine, the quest for pain control during dermatologic procedures has been a constant endeavor [1]. This manuscript delves into the historical journey of dermatological anesthesia, exploring the ingenuity and perseverance of ancient civilizations in their pursuit of pain relief. Through the ages, various methods were employed, ranging from herbal concoctions and compression techniques to the discovery of cold as an anesthetic agent [1,2]. The introduction of anesthesia revolutionized the field of surgery and opened the door to more complex and life-saving procedures. This study traces the evolution of dermatological anesthesia, from its ancient origins to the modern era, highlighting the pivotal discoveries that have paved the way for painless and minimally invasive procedures. By delving into this historical trajectory, we gain insights into the remarkable advancements that have improved patient comfort and transformed dermatologic practice. As we continue to push the boundaries of medical research, the quest for even more efficacious methods of dermatological anesthesia promises a future of continued progress and enhanced patient care.

## Review

Materials and methods

The investigation into the history of dermatological anesthesia involved an extensive literature review, encompassing historical texts, medical manuscripts, and academic publications spanning various periods and civilizations. The aim was to trace the evolution of dermatological anesthesia from ancient times to the modern era, exploring the techniques and substances used for pain relief during dermatologic procedures.

A systematic search was conducted across multiple databases, including PubMed and Embase. Furthermore, Google and historical medical archives were used to identify relevant historical sources and academic articles related to the use of anesthesia in dermatologic procedures. Information from the identified sources was carefully extracted, and historical evidence of anesthesia practices in different civilizations was compiled. Key elements, such as the substances used, methods of administration, and associated outcomes, were analyzed to understand the effectiveness and evolution of dermatological anesthesia over time.

For the medical databases, Pubmed and Embase, as well as the Google search engine, sources meeting the inclusion criteria were those that offered comprehensive descriptions of dermatological anesthesia methods. They also needed to offer historical context, linking the described anesthesia methods to specific periods or civilizations. In addition to historical sources, articles discussing current and up-to-date anesthesia practices were also included. Sources lacking sufficient details regarding dermatological anesthesia methods were excluded and so were texts unrelated to the history of dermatological anesthesia. This process yielded a total of 16 resources, a combination of articles and websites (Table 1).

**Table 1:** Reports used in this study. This study references articles sourced from PubMed and Embase databases. Relevant materials were also obtained from the Google search engine, including articles and websites.

Article	Summary
Lutnick et al. [1]	Delves into the historic perspectives of circumcision and anesthesia, offering insights from the Journal of Urology.
Science Museum [2]	Discusses the art of anesthesia, showcasing its historical significance and developments.
Singh [3]	The article highlights Sushruta's contributions as the father of surgery, providing insight into ancient surgical practices.
Davison [4]	The paper discusses the evolution of anesthesia, shedding light on the advancements made in the field throughout history.
Chenwei Medical [5]	Offers a webpage dedicated to the history of anesthesia, providing insights into its evolution over time.
Kaur et al. [6]	Focuses on achieving hemostasis in dermatology procedures and provides management guidelines.
Science Museum Group Collection [7]	The site explains the invention of the Richardson Spray invented by Benjamin Ward used to spray ether for local anesthesia during tooth extraction, and later adapted by Joseph Lister for use in antisepsis, making it the only surviving spray of its kind.
Marion and Gibbons [8]	Discusses the use of cocaine as a local anesthetic in dermatology, highlighting its relevance.
Nathan et al. [9]	Explains early work on local anesthesia, considered instrumental in its development. Provides historical context and references.
Grant and Hoffman [10]	Discusses the use of topical anesthetics in emergency medicine.
Rękas-Dudziak et al. [11]	Categorizes local anesthetics into esters and amides, detailing their chemical properties and mechanisms of action. Bupivacaine and ropivacaine are also discussed, highlighting their applications and safety profiles.
Upadya and Upadya​​​​​​​ [12]	Provides an updated review of local anesthetic drugs that are used to conduct dermatological surgical procedures.
Crisan et al. [13]	This article examines the existing information on pain relief, anesthesia, and potential complications in pediatric dermatosurgery. The insights from this review can be valuable for enhancing safety and care quality, as well as providing better guidance for parents.
Roerden et al. [14]	Discusses the uses of tumescent local anesthesia (TLA) and its benefits (prolonged anesthesia, reduced bleeding, and avoidance of complications associated) over general anesthesia in infants.
Kouba et al. [15]	Addresses key clinical queries on the use and safety of local anesthesia in dermatologic office-based procedures. It aims to optimize safety and care quality while providing recommendations for dermatologists.
Epstein et al. [16]	This manuscript discusses pain management during laser therapy for cutaneous lesions. Topical anesthesia, nerve blocks, oral sedation, intravenous sedation, and general anesthesia are all discussed as possible modalities.

**Figure 1:**
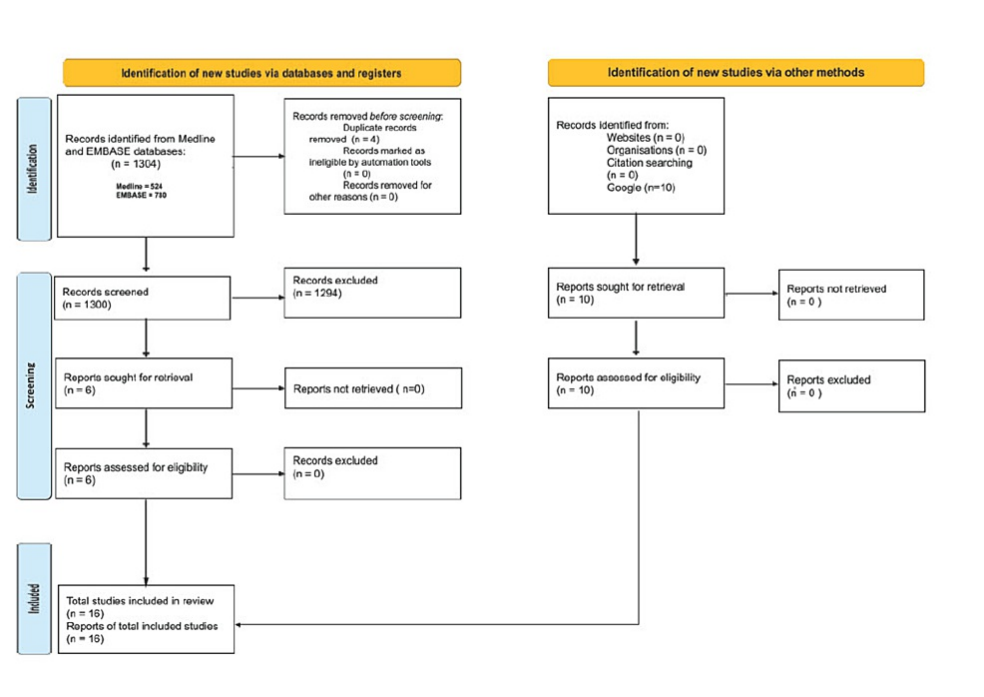
PRISMA study flow chart. PRISMA: Preferred Reporting Items for Systematic Reviews and Meta-Analyses

To identify potentially pertinent articles for our research, we employed the Preferred Reporting Items for Systematic Reviews and Meta-Analyses (PRISMA) systematic review approach [17]. We conducted an extensive search on the PubMed (Medline) and Embase databases to acquire relevant articles. In step 1, we employed a comprehensive keyword search using the phrase "Dermatology Anesthesia Surgery." The total number of articles these search phrases generated was 524 for PubMed and 780 for Embase. The number of articles that appeared from each search phrase is shown in Figure 1. These articles encompass a period from 1969 to 2023. Before the articles were screened, eight duplicates were removed. The criteria for inclusion was whether or not the article included information about the history of dermatologic anesthesia based on title and abstract. If the article contained relevant information, then the whole study was analyzed to determine if it should be used. Additional relevant articles were added through the World Wide Web. The whole process yielded qualitative information that amounted to a total of 16 relevant articles for our topic of interest. These articles were examined in detail, wherein the results and descriptions were extracted from the study.

Results

To investigate the history of dermatologic anesthesia is to delve into the history of human suffering and pain, tracing their origins back to ancient times. One of the first recorded uses of this anesthesia was amongst the ancient Egyptians during circumcision procedures. Although the forced practice of circumcision on captured enemies of the Egyptians traces back to the 23rd century BC, it is unlikely that any anesthesia was used at that time. However, circumcision gradually became adopted by the nobility in ancient Egypt as a rite of passage into the priesthood, as depicted by the illustrations in the sarcophagus of Ankh-maHor. It was not until 2500 BC that the Egyptians then introduced the use of anesthesia during circumcision [1]. It is speculated that they used a concoction of carbonates from limestone and acid which, upon contact, would release carbon dioxide and subsequently act as a local anesthetic.

In 400 BC, an alternate method of anesthesia developed by the Assyrians for circumcision was bilateral carotid artery compression. The compression of the common carotid arteries would induce syncope and decrease pain immediately prior to the procedure. It is likely that other civilizations were engaging in this practice, as the Greeks and Russians termed the carotid artery the “Artery of Sleep.”

Although the surgical field has advanced astronomically, many of its doctrines can be traced back to ancient Indian scholars. In the fifth century BC, Sushruta, widely regarded as the father of plastic surgery, described eight types of surgery: Chedya (excision), Lekhya (scarification), Vedhya (puncturing), Esya (exploration), Ahrya (extraction), Vsraya (evacuation), and Sivya (suturing) [3]. During such procedures, he induced anesthesia using materials such as wine or henbane.

In the 11th century AD, the next promising dermatologic anesthetic was brought forth: cold. An unknown Anglo-Saxon monk discovered the analgesic properties of applying cold water to an area of skin and detailed his findings in the Lacnunga, a collection of medical texts and prayers. Within these texts, cold therapy was apparently used to treat “eruptive rash,” wherein once adequately anesthetized, one could “cut four scarifications around the pocks” to surgically remove the rash [4].

Many of the following innovations in dermatologic anesthesia were refinements and enhancements of already well-documented methods. By the 1500s, a concoction of henbane, opium, and mandragora was the anesthetic of choice for surgery, with their continued use into the 19th century [4]. In 1784, James Moore, a London surgeon, published literature regarding anesthesia induced by direct compression of nerves rather than the carotid arteries. He encouraged the use of clamps for nerve and limb compression [5]. This method is used even today to achieve anesthesia and hemostasis in smaller areas of the body, such as the mucosal lip, nose, and external ear [6].

In 1866, Benjamin Ward Richardson invented the ether spray. Originally intended to be used with a highly volatile hydrocarbon called rhigolene, it was used with ether instead. This was convenient in that the spray allowed the solution to be applied directly to a desired area [7]. It could be administered topically or in deeper incisions and was particularly useful in the opening of abscesses. Not long after in 1884, Sigmund Freud started experimenting with cocaine on the cornea of dogs and guinea pigs, realizing that it could be used as an anesthetic [8]. The use of cocaine as local anesthesia then spread rapidly throughout Europe and America. However, the non-standard dosing led to increasing reports of unintended tachycardia, excitability, mydriasis, and increased sympathetic tone. Its widespread use also resulted in a rising incidence of cocaine addiction amongst medical staff.

Some physicians and surgeons were reluctant to use cocaine due to its systemic toxicity and addictive potential. However, in 1897 Heinrich Braun demonstrated that the toxicity of cocaine was directly correlated to its rate of absorption. He subsequently found that the use of epinephrine along with cocaine achieved long-lasting anesthesia confined to the injection site while minimizing the systemic side effects [9]. Not only did this minimize the potential toxicity of cocaine, but it also allowed for it to serve as an effective local anesthetic. This formula is still currently used when suturing skin lacerations in the pediatric population [10].

Discoveries in organic chemistry made in the late 19th and early 20th centuries resulted in the ability to isolate pure cocaine and create new local anesthetics. Among these new local anesthetics were the ester anesthetics, such as procaine, holocaine, orthoform, benzocaine, and tetracaine. These medications are comprised of hydrophilic particles that combine a tertiary amine with a lipophilic aromatic ring [11]. When in their ionized states, their mechanism of action involves blocking rapid sodium channels found in neuronal cell membranes. The invention of procaine by Alfred Einhorn was a pivotal point in the history of local anesthesia because when compared to cocaine, the drug has a significantly lower side effect profile along with diminished addictive potential. For this reason, procaine quickly became the prototypical local anesthetic soon after its invention in 1905, just a year after Braun combined the use of epinephrine and cocaine. Nevertheless, these ester anesthetics often led to the occurrence of contact dermatitis [11].

Shortly following the esters were the amino amide local anesthetics, such as chloroprocaine, cinchocaine, lidocaine, mepivacaine, and prilocaine. Amides, like esters, are tertiary amines that work through a similar mechanism [11]. Lidocaine, the first non-ester anesthetic, quickly became a widely used agent in dermatologic procedures due to its increased potency and low side effect profile. Currently, lidocaine with epinephrine is the most common anesthetic for local infiltration due to its rapid onset and moderate duration of action. It is also available as Eutectic Mixture of Local Anesthetics (EMLA), an oil-water emulsion of 2.5% lidocaine and 2.5% prilocaine with polyoxyethylene fatty acid emulsifier enhancing absorption. Lidocaine 4% is also available in the form of a gel and is also good for surface anesthesia [12]. Bupivacaine with epinephrine is another amide anesthetic that has been widely adopted, primarily because of its longer onset and duration of action than lidocaine. Today, bupivacaine is used for epidural anesthesia and as regional blocker [11].

With the current advancements in modern medicine, many patients expect to have dermatological and aesthetic procedures that are painless and minimally invasive. Today local, superficial anesthesia is administered via the two following primary methods: the use of topical anesthetics and administration with needles. Topical anesthetics are notably used for dermatologic procedures to anesthetize mucous membranes within the skin. The maximum effect of these anesthetics, usually lidocaine, takes effect within 5 minutes and can last for up to an hour. However, a disadvantage of these topical anesthetics is the difficulty of dosing the anesthetic.

Without the invention and evolution of dermatologic anesthesia, many procedures could not be adequately performed due to their inherent painful nature. It is evident that dermatologic anesthesia has advanced significantly since its inception in achieving necessary analgesia and patient comfort. With further research, dermatologic procedural pain control will undoubtedly continue to progress by refining already established methods or developing entirely novel, but more efficacious medications.

Discussion

The investigation into the history of dermatological anesthesia offers valuable insights into the evolution of pain relief practices during dermatologic procedures from ancient civilizations to the present day. The findings shed light on the remarkable ingenuity and perseverance of early societies in alleviating human suffering and highlight the significant milestones that have paved the way for modern medical practices.

Clinical Implications

The historical journey of dermatological anesthesia demonstrates the critical role of anesthesia in modern medical practices. From the ancient origins of pain relief to the refined techniques and formulations of today, the quest for patient comfort and safety has remained at the forefront of medical innovation. The lessons learned from historical practices have significantly contributed to the improvement of contemporary dermatologic procedures, making them more accessible, less invasive, and less painful for patients.

Pediatric application: Dermatologic surgery in infants and children is on the rise, driven by the proportional increase in the size of skin lesions with age [13]. The early removal of these lesions is emphasized for its positive impact on cosmetic outcomes, capitalizing on enhanced skin elasticity and robust tissue regeneration. EMLA, and other local anesthetics in pediatric dermatosurgery, particularly for excising conditions like congenital and sebaceous nevi, Spitz nevi, acquired melanocytic nevi, and dermoid or epidermal cysts [14]. The manuscript highlights the significance of lidocaine and its formulations in pediatric dermatosurgical procedures. EMLA, a eutectic mixture of lidocaine and prilocaine, is discussed, underscoring its efficacy when applied to intact skin under occlusion for achieving dermal analgesia [12,13].

Cutaneous laser procedures: The role of dermatologic anesthesia in cutaneous laser therapy is crucial, and various methods are employed to manage pain and discomfort during these procedures. In dermatology, topical anesthesia is a frequently employed approach, particularly in cutaneous laser procedures. Topical agents are utilized to achieve anesthesia that proves effective for a range of non-ablative laser treatments [15]. Some expert experiences and limited literature data suggest that for certain patients, topical anesthesia alone may be adequate, even for ablative laser resurfacing. However, more commonly, topical anesthesia is used in conjunction with infiltrative anesthesia or cutaneous nerve blocks during ablative procedures [15,3]. This combination of anesthesia methods is aimed at ensuring patient comfort and minimizing pain associated with laser therapy, taking into account individual variations in pain tolerance and the specific characteristics of the cutaneous lesions being treated. There is an emphasis on an individualized approach to pain management in cutaneous laser therapy, particularly in pediatric patients with port-wine stains, where the choice of anesthesia may vary based on factors such as lesion size, location, and patient age [3]. Overall, the goal is to provide effective analgesia while considering the unique challenges posed by cutaneous laser procedures, such as the risk of fire during treatment, especially under general anesthesia. Collaboration with an anesthesiologist is recommended for patients requiring more intensive forms of analgesia or sedation.

Tumescent local anesthesia: Tumescent local anesthesia (TLA), originally developed by a dermatologist, involves subcutaneous infiltration of large volumes of dilute anesthetic, inducing swelling and firmness in targeted areas [15]. This technique, reinvented in 1987 for liposuction surgery, has expanded into various fields, including pediatric dermatologic surgery as highlighted by Roerden et al. [14]. TLA in infants has shown benefits such as excellent pain control, long-lasting analgesia, and reduced bleeding risk. Despite concerns about epinephrine, its use in TLA has demonstrated safety. Dosing guidelines vary, but some recommend maximum dosages for specific local anesthetics in pediatric settings. The manuscript underscores the importance of adequate pain control in infancy and asserts that TLA is generally safe and convenient for pediatric surgery, although further studies are needed to establish guidelines and identify potential complications in this population.

There is substantial evidence supporting the safety of tumescent local anesthesia (TLA) when used for office-based liposuction [15]. Kouba et al. estimate the rate of serious adverse events to be 0.04-0.16%, with no reports of death associated with liposuction performed under TLA by dermatologists. Lidocaine with epinephrine, the most commonly studied solution, has been shown to be effective at various concentrations. A well-conducted prospective study found local tumescent anesthesia with lidocaine doses of 55 mg/kg to be safe for use in office-based liposuction, and expert experience supports this finding. The manuscript emphasizes the safety and efficacy of TLA, particularly in liposuction, and highlights its expanding applications.

Dosing:* *The administration of local infiltrative anesthesia during dermatologic procedures necessitates a nuanced understanding of dosages and potential toxic effects. While the maximum safe dose remains unknown, manufacturer-recommended limits guide lidocaine administration, indicating 7 mg/kg with epinephrine and 4.5 mg/kg without epinephrine for adults [15]. In the pediatric context, doses of 3.0-4.5 mg/kg with epinephrine and 1.5-2.0 mg/kg without epinephrine are considered safe based on expert opinion and clinical experience. However, the absence of published evidence supporting these limits underscores the need for further research in this area.

Tumescent local anesthesia, a specialized form, introduces additional considerations [15]. Safety parameters for office-based liposuction using tumescent local anesthesia suggest a total lidocaine dose of 55 mg/kg. Incremental dosing studies remain sparse, with a single prospective cohort study indicating the safety of 50 mL of 1% lidocaine delivered incrementally over an average of 8 hours for Mohs Micrographic Surgery (MMS).

In practices for diseases of the head and neck, and beyond, a corrective agent, usually adrenaline, is added to the anesthetic, commonly lidocaine, in a ratio of 1:100000. This aims to induce vasospasm in small blood vessels (capillaries), resulting in two following positive outcomes for both the doctor and the patient: (1) prolonged anesthetic effect, nearly doubling the pain relief duration, even after operative techniques following general anesthesia; and (2) improved hemostasis, leading to reduced blood loss during the surgical intervention [18-21].

Toxicity prevention: Toxicity monitoring and prevention strategies are paramount, particularly in the context of local anesthetic systemic toxicity (LAST), which exhibits lasting interpatient variability and typically progresses through central nervous system excitement [15]. Recognizing early signs, such as circumoral numbness and altered speech, is crucial. While dermatologic procedures generally involve doses well below manufacturer-recommended maximums, vigilance is essential. A formula for calculating the maximum allowable volume of common local anesthetics aids in preventing calculation errors.

Despite the rarity of anesthetic toxicity in dermatologic settings, clinicians should remain mindful. Recommended steps for toxicity prevention include awareness of potential signs, calculations of maximum allowable volume, and consideration of ultrasonographic guidance and intravascular markers, although the latter may be impractical for routine dermatologic use. The detailed overview underscores the importance of meticulous dosing and vigilant toxicity monitoring during dermatologic anesthesia procedures. Caution is particularly warranted in specific populations, such as infants below three months of age, where there is an increased risk of methemoglobinemia associated with EMLA [14].

Current Challenges and Future Prospects

Despite the remarkable progress in dermatological anesthesia, challenges remain in dosing and precision administration of topical anesthetics. Efforts are ongoing to enhance the effectiveness and duration of anesthesia while minimizing potential side effects. Continued research and advancements in medical science hold promise for the development of more efficacious and targeted anesthetic medications.

For example, local skin anesthesia plays a crucial role in the relief of specific diseases, such as auriculotemporal syndrome (Fray's syndrome). This syndrome is characterized by aberrations between the branches of the auriculotemporal nerve and the greater auricular nerve, causing patients to turn red and sweat in the area before and below the ear when eating. Administering local anesthesia, which involves blocking the firing of nerve impulses of the aberrant nerves, can temporarily prevent this condition that significantly worsens the quality of life [22].

Limitations

One limitation of this study was that only two medical databases, PubMed and Embase, were incorporated to identify six articles relevant to this review. Although Google was used to supplement additional sources, a few of them were websites that had not undergone the rigorous peer-review process. Moreover, this systematic review primarily incorporated sources such as literature reviews, narrative reviews, and expert opinions. As a result, risk of bias assessments were not conducted, as these studies did not encompass methods such as study design, statistical analyses, participant selection, randomized control trials, and similar elements.

## Conclusions

The exploration of the history of dermatological anesthesia reveals a fascinating timeline of human ingenuity in mitigating pain during medical interventions. Early civilizations' innovations have laid the foundation for today's sophisticated and safe anesthesia practices. It is therefore evident that the quest for enhanced pain control in dermatology is not merely a historical narrative but an ongoing endeavor.

As medical knowledge continues to expand, the journey to improve patient comfort and optimize dermatologic procedures remains an ongoing endeavor. The future holds promise for even more efficacious and patient-centered pain control measures in the field of dermatology.
